# Asexual reproduction induces a rapid and permanent loss of sexual reproduction capacity in the rice fungal pathogen *Magnaporthe oryzae*: results of *in vitro *experimental evolution assays

**DOI:** 10.1186/1471-2148-12-42

**Published:** 2012-03-29

**Authors:** Dounia Saleh, Joëlle Milazzo, Henri Adreit, Didier Tharreau, Elisabeth Fournier

**Affiliations:** 1 INRA, UMR BGPI, TA A54/K, 34398 Montpellier, France; 2 CIRAD, UMR BGPI, TA A54/K, 34398 Montpellier, France

## Abstract

**Background:**

Sexual reproduction is common in eukaryotic microorganisms, with few species reproducing exclusively asexually. However, in some organisms, such as fungi, asexual reproduction alternates with episodic sexual reproduction events. Fungi are thus appropriate organisms for studies of the reasons for the selection of sexuality or clonality and of the mechanisms underlying this selection. *Magnaporthe oryzae*, an Ascomycete causing blast disease on rice, reproduces mostly asexually *in natura*. Sexual reproduction is possible *in vitro *and requires (i) two strains of opposite mating types including (ii) at least one female-fertile strain (*i.e*. a strain able to produce perithecia, the female organs in which meiosis occurs). Female-fertile strains are found only in limited areas of Asia, in which evidence for contemporary recombination has recently been obtained. We induced the forced evolution of four Chinese female-fertile strains *in vitro *by the weekly transfer of asexual spores (conidia) between Petri dishes. We aimed to determine whether female fertility was rapidly lost in the absence of sexual reproduction and whether this loss was controlled genetically or epigenetically.

**Results:**

All the strains became female-sterile after 10 to 19 rounds of selection under asexual conditions. As no single-spore isolation was carried out, the observed decrease in the production of perithecia reflected the emergence and the invasion of female-sterile mutants. The female-sterile phenotype segregated in the offspring of crosses between female-sterile evolved strains and female-fertile wild-type strains. This segregation was maintained in the second generation in backcrosses. Female-sterile evolved strains were subjected to several stresses, but none induced the restoration of female fertility. This loss of fertility was therefore probably due to genetic rather than epigenetic mechanisms. In competition experiments, female-sterile mutants produced similar numbers of viable conidia to wild-type strains, but released them more efficiently. This advantage may account for the invasion of our populations by female-sterile mutants.

**Conclusions:**

We show for the first time that, in the absence of sexual reproduction, female-sterile mutants of *M. oryzae *rice strains can arise and increase in abundance in asexual generations. This change in phenotype was frequent and probably caused by mutation. These results suggest that female fertility may have been lost rapidly during the dispersion of the fungus from Asia to the rest of the world.

## Background

Sexual reproduction is a widespread mode of reproduction in eukaryotic microorganisms, only a small proportion of which reproduce exclusively asexually [1]. However, sexual and asexual modes of reproduction are not exclusive in some species. Episodes of sexual reproduction may occur, for example, in fungal species that mostly reproduce asexually [2,3]. Furthermore, it has recently been shown that many fungal species previously thought to be clonal may also reproduce sexually [4]. For example, Campbell and Carter [5] demonstrated the occurrence of recombination in *Cryptococcus neoformans *and *C. gattii*. O'Gorman *et al. *[6] recently discovered a sexual cycle *in vitro *in *Aspergillus fumigatus*. Seidl *et al. *[7] identified mating types and provided the first evidence for the induction of sexual reproduction in *Trichoderma reesei*. In total, about 55,000 of the 70,000 known fungal species have been shown to reproduce both sexually and asexually [3], and sexual reproduction probably occurs in many of the other species but has yet to be detected [8]. Moreover, evolutionary shifts from sexual to asexual reproduction or *vice versa*, have been observed in related species of fungi, in the genera *Penicillium *[9], *Microsporidia *[10], *Aspergillus *[11] and *Candida *[12], for example. Fungi are therefore good model organisms for studies of how and why a particular mode of reproduction is selected.

The modes of reproduction observed among sexually reproducing fungi are diverse [13,14]. Sexual reproduction is based on the recognition of opposite mating types through a pheromone/receptor system, triggering cell-cell fusion followed by meiosis. Some fungi are heterothallic, with different individuals having opposite mating types, rendering outcrossing obligatory. Conversely, in homothallic fungi, both mating types are encoded by the same genome, and an individual can mate with any other individual, including itself [15]. Same-sex mating -- a given individual being able to mate with itself despite carrying only one mating type -- has also been demonstrated in some heterothallic species, such as *Candida albicans *[16]. Another key component of sexual reproduction in several fungi is the ability to produce the female organs in which meiosis takes place. This ability is referred to as "female fertility", and sexual reproduction is not possible in the absence of a female-fertile strain. In addition to the MAT genes responsible for determining mating type, many loci with quantitative and pleiotropic effects, including a number involved in regulatory pathways, have been shown to contribute to sexual reproduction and, in particular, to the differentiation of female organs [17]. Mutations responsible for female-sterile phenotypes have already been identified in such loci in fungi: *Agaricus bisporus *[18], *Podospora sp. *[19], *Fusarium sp*. [20], *Neurospora crassa *[21], *Podospora anserina *[22,23]. Epigenetic effects may also be involved in sterile phenotypes [24]. This multifactorial control of sexual reproduction may result in its rapid loss due to mutations or changes in the expression of any of the genes involved [25]. However, there are few experimental data supporting the prediction of a rapid loss of female fertility following a period of exclusive asexual reproduction in a species normally displaying both sexual and asexual reproduction. Xu [17] reported a complete loss of the ability to reproduce sexually in two strains of *C. neoformans *after several generations of asexual reproduction *in vitro*. They estimated the associated minimum genome-wide mutation rates for these two strains to be 0.0172 and 0.0772. However, they did not determine whether genetic or epigenetic mechanisms were responsible for the emergence of this phenotype.

In this study, we investigated whether there was selection against female fertility in the absence of sexual reproduction events. We investigated whether female fertility was lost under conditions of strict asexual reproduction and whether female-sterile mutants had a fitness advantage over female-fertile wild-type strains. In fungi, fitness--the ability to survive and reproduce--is related to several traits [26], including growth, asexual sporulation rates and the number of viable asexual spores released. We measured these traits separately in female-fertile wild-type strains and female-sterile evolved strains generated by experimental evolution, and evaluated comparative fitness by following these traits in competition experiments [27]. We used the fungal species *Magnaporthe oryzae*, for which sexual reproduction has been clearly demonstrated. This Ascomycete causes the most important fungal disease of cultivated rice worldwide, blast [28,29].

In *M. oryzae*, the pathogenic cycle observed in the field is asexual. The rice blast fungus attacks all aerial parts of the plant. Leaf infection is initiated by the attachment of asexual spores (conidia) to the rice leaf cuticle [30]. The conidia produce an appressorium, which penetrates host tissues, forming a germ tube growing into the host cell. The fungus produces a mycelium and colonises the host tissue [30]. Lesions appear after five to seven days and, in humid conditions, conidiophores and conidia are produced outside the plant, initiating a new cycle. The sexual stage has never been observed in the field. However, some strains from the centre of origin of this fungus (the Himalayan foothills) are able to reproduce sexually *in vitro *and population genetics studies have shown that sexual reproduction continues to occur, at least in limited areas, within this region [31]. *M. oryzae *is a heterothallic species. The two mating types, Mat1.1 and Mat1.2, are determined by two idiomorphs on chromosome 7 [32]. For sexual reproduction to occur between two strains, at least one of the strains involved, regardless of mating type, must be able to produce perithecia (female-fertile). Female-fertile strains are rare and are found almost exclusively close to the putative centre of origin of the fungus [33]. This species thus includes populations from the centre of origin in which individuals display an alternation of sexual and asexual reproduction and populations from elsewhere that display exclusively clonal reproduction. The most parsimonious hypothesis is that *M. oryzae *may have lost the ability to reproduce sexually during its spread from the centre of origin to the rest of the world [31]. *M. oryzae *is thus an appropriate biological model for studying the loss of sexual reproduction capacity within a given species. Moreover, a loss of female fertility has already been observed *in vitro *in *M. oryzae *isolates from finger millet (*Eleusine coracana*) [34]. Our objective was thus to determine whether rice strains of *M. oryzae *lost their female fertility in the absence of sexual reproduction and to determine whether this loss was controlled by genetic or epigenetic factors. We monitored changes in female fertility in the absence of sexual reproduction in an *in vitro *experiment. We then crossed evolved female-sterile strains and wild-type strains, to study the segregation of the female-sterile phenotype in the progeny. We also investigated whether female fertility could be restored by subjecting the evolved female-sterile strains to stresses. Finally, we investigated the possible fitness advantage of female-sterile strains, by analysing several traits related to fitness (growth, asexual sporulation rates, and the number of viable asexual spores released) and comparing them between female-fertile wild-type strains and female-sterile evolved strains from the experimental assay, both separately and in competition experiments.

## Methods

### Collection and storage of strains

We chose four female-fertile strains collected in 2008 from a single rice population in South China (Table 1). In this sample, 19 of the 24 strains were female-fertile and all population genetics indices (genotypic diversity, linkage disequilibrium) were consistent with contemporary sexual reproduction [31].

**Table 1:** Strains used for the experiment, mating type, experimental design and parameters of the Poisson regressions fitted to the data

Last culture	Duration of the experiment ("clonal generations"/days)	*R^2^*		*a*		*b*		*t_50_*		*ρ*	
		
		**1^st ^ref**.	**2^nd ^ref**.	**1^st ^ref**.	**2^nd ^ref**.	**1^st ^ref**.	**2^nd ^ref**.	**1^st ^ref**.	**2^nd ^ref**.	**1^st ^ref**.	**2^nd ^ref**.
S1-A10	10/73	0.03	0.01	4.4	4.9	0.02	-0.01	-	-	-0.65	0.69
S1-B20	20/146	0.82	0.53	4.8	5.1	-0.28	-0.15	2.5	4.5	0.56	0.64

S2-A20	20/146	0.32	0.32	5.4	5.5	-0.08	-0.08	8.6	8.4	0.38	0.60
S2-B20	20/146	0.85	0.94	5.4	5.6	-0.21	-0.24	3.3	2.9	0.82*	0.98*

S3-A10	10/73	0.36	0.67	5.5	5.7	-0.14	-0.20	3.7	3.5	0.64	0.90*
S3-B20	20/146	0.67	0.87	5.6	5.8	-0.19	-0.30	3.7	2.3	0.65	0.82*

S4-A20	20/146	0.46	0.94	5.8	5.4	-0.13	-0.40	5.4	1.7	0.84*	0.95*
S4-B20	20/146	0.60	0.99	5.7	5.6	-0.22	-0.46	3.2	1.5	0.89*	0.85*

Two replicates were performed for each strain (A and B). The name of experimental strains is given as Sx-R_AG_, where Sx is the name of the original wild-type strain, R is the replicate (A or B), and AG is the number of "asexual generations". The duration of the experiment is given in number of AGs and number of days. The "intercept" (*a*) and "slope" (*b*) of the Poisson regressions *y *= *e^a+bt ^*(*y*: number of perithecia produced by the evolved strain, *t *= number of AGs) and *t_50 _*are given for each evolved strain with the two reference strains (1^st ^and 2^nd ^ref.). *R^2^*: Coefficient of determination for each adjustment. *ρ*: Pearson's coefficient for the correlation between female fertility (= number of perithecia produced) and male fertility (= number of perithecia induced) for each strain against each reference strain in each replicate (* indicates values significantly different from 0, test for association between paired samples)

Two of the chosen strains were Mat1.1 (CH999, hereafter named S1, and CH1003, hereafter named S2) and the other two were Mat1.2 (CH997, hereafter named S3, and CH1019 hereafter named S4). All the experiments (strain cultures and sexual crosses, see below) were performed on rice flour agar medium (20 g rice flour, 2 g yeast extract, 15 g agar and 1 L water, with the addition of 500 000 IU of penicillin G after autoclaving for 20 min at 120°C).

### Experimental evolution

We generated two replicates (A and B) of experimental evolution for each of the four strains. At the start of the experiment, each strain was grown at 25°C on rice flour agar medium in a Petri dish with a diameter of 90 mm. After seven days, the culture was subjected to two parallel treatments:

(i) Storage at -20°C on dried filter paper, as described by Valent *et al. *[35]. No single-spore isolation was carried out.

(ii) Transfer of asexual spores (conidia) to a new 90 mm Petri dish, to initiate the next culture. This transfer was accomplished by inverting the initial Petri dish and stamping it onto a new dish.

This procedure was repeated consecutively 10 to 20 times. The "asexual generation" (AG) is defined as the seven-day period separating two consecutive transfers. An AG encompassed at least 220 mitotic multiplication cycles, as estimated by dividing the distance colonized on a Petri dish in one AG (2.2 cm) by the mean size of a mycelium cell (100 μm). For each replicate of each strain, we obtained 10 to 20 experimental strains, one per AG. The evolved strains were named as follows: Sx-R_AG_, where Sx is the name of the original wild-type strain, R is the replicate (A or B), and AG is the number of asexual generations. For example, S3-A_5 _was the strain evolved from S3 during five AGs in replicate A.

At the end of the experiment, fresh cultures of all evolved strains were generated from the stocks obtained in each AG. These cultures were used to evaluate the female fertility of each evolved strain, by testing against reference strains of known mating types, as described below.

### Fertility assessment

*M. oryzae *can reproduce sexually when mycelia from strains of opposite mating types come into contact, if at least one of the strains is female-fertile. The structures involved in fertilisation in *M. oryzae *have not yet been clearly identified. Microconidia are rarely observed and are thought to be male elements, but no role in sexual reproduction has ever been demonstrated for these spores [36,37]. Conidia do not seem to be involved in sexual reproduction, because crosses between two aconidial mutants resulted in a complete sexual cycle [38]. In *M. oryzae*, meiosis is immediately followed by a single mitotic division to generate four pairs of sister ascospores grouped into a single ascus. The ascospores are formed within the sexual structures -- the perithecia -- produced by the female-fertile strain (or by both strains if both are female-fertile).

**Figure 1:**
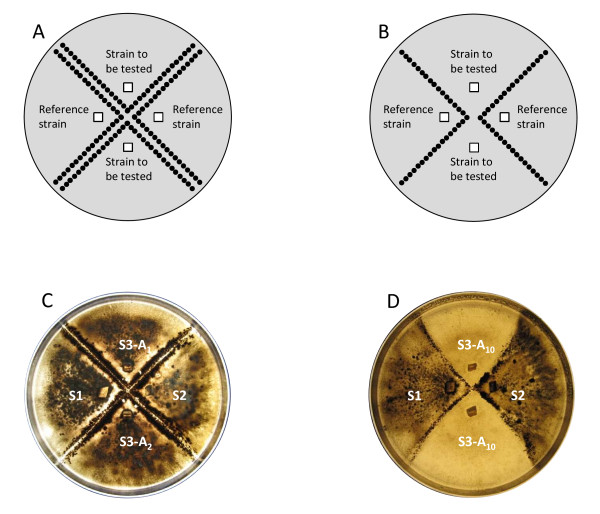
***Magnaporthe oryzae *sexual crosses on rice flour agar medium**. Design of the crosses and schematic diagram of results (A and B). A. Two lines of perithecia are produced between each tested strain and each reference strain: the tested strains are female-fertile and male-fertile. B. A single line of perithecia is produced between each tested strain and each reference strain: the tested strains are female-sterile and male-fertile. C and D: crosses of evolved strains derived from S3 at three different asexual generations (1, 2 and 10) with the two reference strains S1 and S2. C. Tests with S3-A_1 _and S3-A_2_: both strains are female-fertile. D. Tests with S3-A_10 _(same strain tested twice): the strain is female-sterile.

We assessed the female fertility of the evolved strains, by evaluating their ability to produce perithecia in the presence of reference strains. Reference strains are hermaphrodite strains of known mating type that produce large numbers of perithecia when crossed with a strain of the opposite mating type (Figure 1A, B). We used the four wild-type strains as reference strains in crosses with evolved strains. For the strains evolved from S1 and S2 (Mat1.1), we used both S3 and S4 (Mat1.2) as the reference strains. For the strains evolved from S3 and S4 (Mat1.2), we used both S1 and S2 (Mat1.1) as the reference strains. These crosses also made it possible to evaluate the male fertility of each of the evolved strains: its ability to induce the production of perithecia by the reference strains.

Crosses were performed as described by Nottéghem and Silué [39], on rice flour agar medium in 90 mm Petri dishes. Cultures were incubated for two days at 25°C and were then placed under continuous light at 20°C. The production of perithecia was observed after 21 days of incubation in these conditions. The formation of two rows of perithecia between a tested strain and a reference strain indicated that both strains were male-fertile and female-fertile (Figure 1C). The observation of a single row indicated that only the reference strain produced perithecia, and that the tested strain was female-sterile but male-fertile (Figure 1D). An absence of perithecia indicated that the tested strain was both female-sterile and male-sterile. Crosses between each wild-type strain and the reference strains of opposite mating type were used as positive controls for comparison with evolved strains. For each strain and experiment, evolved strains were collected at different AGs and the number of perithecia produced was estimated for each line by counting the perithecia in three 3 × 3 mm squares evenly distributed along the corresponding line of perithecia. If no perithecia were detected in the squares, we checked that no perithecia at all had been formed on the Petri dish.

### Obtaining progenies for genetic studies

We isolated progeny from two crosses between a wild-type strain and an evolved strain that had lost female fertility: S3-A_10 _was crossed with S1 (cross #126) and S1-B_12 _was crossed with S3 (cross #127). For ascospore isolation, 19 to 21 days after crossing (see above), mature perithecia were placed on water-agar medium (40 g Bacto agar in 1 litre of distilled water) and opened with a scalpel to release the asci. The asci were separated with a fine glass needle and, after 20 to 30 min, were gently crushed to release the ascospores. The ascospores were separated with a fine glass needle and incubated at 25°C for 24 h. Germinated ascospores were then transferred to rice flour agar medium and stored as previously described. We retained only one germinated ascospore per ascus, to ensure that each progeny was generated by an independent meiotic event and to prevent the collection of sister ascospores.

### Reprogramming of gene expression in female-sterile evolved strains

We attempted to reverse potential epigenetic effects, by subjecting female-sterile evolved strains to various treatments known to cause or very likely to cause the reprogramming of gene expression [40,41]. We investigated the effects of cold temperature and mycelium fragmentation on S1-B_12 _and S3-A_10 _and on the corresponding wild-type strains S1 and S3 (Table 2). These treatments were harmful enough to be considered as stresses but did not kill the mycelium or the conidia. We also subjected these strains to two other treatments that have been shown to reprogramme gene expression: single-spore isolation and growth on plants (Table 2).

**Table 2:** Stresses performed on female-sterile strains S1-B_12 _and S3-A_10 _and the corresponding wild-type strains S1 and S3

	**Proportion of female-fertile replicates before treatment**				**Proportion of female-fertile replicates after treatments**			
	
**Stress**	**S1**	**S3**	**S1-B_12_**	**S1-A_10_**	**S1**	**S3**	**S1-B_12_**	**S3-A_10_**
	
-80°C (1 h)	5/5	5/5	0/5	0/5	5/5	5/5	0/5	0/5
-80°C (24 h)	5/5	5/5	0/5	0/5	5/5	5/5	0/5	0/5
Mycelium mashing	5/5	5/5	0/5	0/5	5/5	5/5	0/5	0/5
single-spore isolation	1/1	1/1	0/1	0/1	12/12	12/12	0/12	0/10
growth on plant	1/1	1/1	0/1	0/1	7/10	10/10	0/8	0/10

The treatments were: one hour at -80°C, 24 hours at -80°C, mycelium mashing by ultrasound, single-spore isolation and growth on plants. The ratios (number of replicates in which the strain was female-fertile)/(number of replicates in which the strain was female-sterile) are given before and after the various treatments

For each treatment, we collected the mycelium or conidia before and after the treatment and used the collected material to evaluate female fertility as described above. For extreme temperature stress, strains were grown on rice flour medium for one week and then placed at -80°C: half the plates for 1 h and the other half for 24 h. For mycelium fragmentation stress, strains were grown on rice flour medium for one week and the surface of the medium was watered and scrubbed, and the harvested mycelium was placed in tubes. The suspension was subjected to ultrasound for 30 seconds to break the mycelium and a drop of the suspension was placed on rice flour medium for crosses. For single-spore isolation, single conidia were collected and spread on Bacto agar medium with a fine glass needle. The plates were incubated for 24 h, and 10 germinated conidia were collected and used for crosses with reference strains. For growth *in planta*, strains were used to inoculate rice plants, as described below. Infected leaves were collected and placed on a moist filter paper for 24 h, to allow sporulation to occur. Conidia were then collected with a fine glass needle and isolated as described above.

### Asexual sporulation

*In vitro *asexual sporulation was estimated for seven female-sterile evolved strains (S1-B_12_, S2-A_19_, S2-B_12_, S3-A_10_, S3-B_15_, S4-A_19_, and S4-B_10_), and for the corresponding wild-type strains (S1, S2, S3, S4). Two replicates were performed per strain. In an additional experiment, five replicates were used for S1-B_12_, S3-A_10_, S1, and S3. After seven days of growth on rice flour agar medium, conidia were collected by watering the plate and scrubbing the surface of the medium. We counted conidia under the microscope, with a haemocytometer, to determine inoculum density. The area of the Petri dish covered by mycelium, reflecting the speed of growth, was estimated by image analysis with LEICA APPLICATION SUITE version 3.7.0 software. We calculated sporulation rates by dividing conidial counts by this mycelial area. The capacity of conidia to germinate and to form appressoria (the organ essential for host tissue penetration) was assessed for S1-B_12_, S3-A_10_, S1, and S3. Calibrated conidial suspensions were deposited on glass slides in a Petri dish also containing moist filter paper and the proportions of germinating conidia and of conidia forming an appressorium were determined after 24 h at 25°C. Sporulation rates were also calculated for 12 and 15 progenies from cross #126 and cross #127, respectively (two replicates for each progeny). In this experiment, we measured colony diameter with a ruler and evaluated growth by measuring the area of the corresponding disk.

*In planta *asexual sporulation was also evaluated for S1-B_12_, S3-A_10_, S1, and S3. Four trays, each containing about 150 rice plants (variety Maratelli), were placed in the greenhouse for four weeks and the plants were inoculated with conidial suspensions (20 ml of a 25,000 conidia.ml^-1 ^suspension supplemented with 1% gelatin), one strain per tray, as previously described [42]. After one week, the number of lesions and the area they covered were estimated on 15 leaves for each strain. A single lesion was collected from each of the 15 leaves. Lesions were pooled in threes, placed in tubes containing 0.01% Tween in 1 mL water (five replicates) and mixed thoroughly. The concentration of conidia was estimated with a haemocytometer and the rate of sporulation was calculated by dividing conidial counts by the area covered by the lesions.

### Competition between female-sterile and female-fertile strains

We also quantified fitness in experiments assessing competition between female-sterile evolved strains and their corresponding female-fertile wild-type strains. We let the wild-type and evolved strains grow together during one AG. We estimated the proportion of female-fertile and female-sterile progeny among the conidia germinating after transfer to a new Petri dish. We used the female-sterile evolved strains S1-B_20_, S2-A_20_, S3-B_20 _and S4-A_20 _and their respective female-fertile wild-type strains S1, S2, S3 and S4 for this experiment. We prepared eight suspensions of conidia (5000 conidia.ml^-1^) for each of these eight strains. We also prepared four suspensions (5000 conidia.ml^-1^) in which evolved strains were mixed with their corresponding wild-type strain (S1/S1-B_20_, S2/S2-A_20_, S3/S3-B_20 _and S4/S4-B_20_). For each of the 12 strains or mixtures, we deposited 40 μl of the suspension on rice flour agar medium. Two replicates were performed for each strain or mixture. After seven days of growth at 25°C (i.e. one AG), conidia were transferred to a new Petri dish, as described above. Plates were incubated for 24 h at 25°C. We then estimated the number of conidia transferred by counting, in three disks of 3 mm diameter. For female-fertile strains grown alone, the results obtained provided an estimate of the minimum number of conidia transferred at the end of an AG. We multiplied the mean number of conidia counted on the three disks by the area of a Petri dish (6359 mm^2^) and divided by the area of a disk (7 mm^2^). We calculated that, on average, for the parental strains S1, S2, and S4, a minimum of 27,000 (± 24 000) conidia were transferred between Petri dishes at each generation. We also recorded the number of germinated conidia. The values were averaged over the three counted disks and over the two replicates. For mixtures of female-sterile and wild-type strains, we collected 20 germinated conidia. Their female-fertile or female-sterile phenotype was determined by crossing them with the appropriate reference strains. This provided an estimate of the proportion of female-fertile germinated conidia (i.e. produced by the wild-type strain) *versus *female-sterile germinated conidia (i.e. produced by the evolved strain) that had been transferred. The values were averaged over the two replicates.

### Statistical analyses

Statistical analyses were performed with R software. For each evolved strain, the number of perithecia (*y*) produced (female fertility) and the number of perithecia induced in the reference strains (male fertility) were plotted as a function of the number of clonal generations (*t*). We fitted Poisson regressions (*y *= *e^a+bt^*) to female fertility and male fertility rather than comparing with the initial values. This method was the most appropriate for taking into account the considerable variation of perithecium production in the first three AG. The number of AG until the evolved strains produced half of the initial number of perithecia (*t_50_*) was estimated for each fit to female fertility curves. The putative effects of mating type (Mat1.1 and Mat1.2), wild-type strain (S1, S2, S3 and S4), replicate (A and B), and reference strain on *t_50 _*were assessed by analysis of variance (ANOVA). *In vitro *and *in planta *differences in asexual sporulation and differences in the area of the mycelium on Petri dishes between the evolved strains S1-B_12 _and S3-A_10 _and the corresponding wild-type strains, S1 and S3, were assessed in Kruskall-Wallis non parametric tests. Departures from expected segregations or ratios were tested in *χ*^2 ^tests.

## Results

### Loss of female fertility

The aim of the experiment was to study changes in female fertility over several generations of asexual reproduction. "Asexual generation" (AG) was defined as the mean period of time between two consecutive episodes of asexual spore production. We calculated that an AG encompassed at least 220 mitotic multiplication cycles (see methods). We crossed the evolving strains at different asexual generations (AG), to estimate the proportion of perithecia produced.

As expected, the level of female fertility observed at *t_0 _*for each wild-type strain depended on the reference strain used for crosses. For example, S3 produced twice as many perithecia when faced with S1 than when faced with S2. This may reflect specificities in the interactions between some strains.

**Figure 2:**
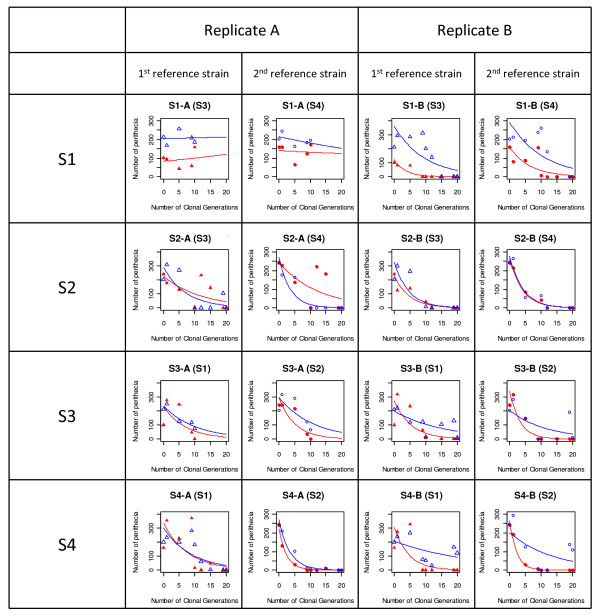
**Estimation of the number of perithecia produced during crosses between the strains after several asexual generations (AGs)**. Perithecia were counted on 9 mm^2 ^squares at three different positions along the confrontation line. Closed red symbols: number of perithecia produced by the evolved strain (female fertility). Open blue symbols: number of perithecia produced by the reference strain, i.e. induced by the evolved strains (male fertility). Triangles: first reference strain; Circles: second reference strain. The name of the reference strain used for the crosses is given in brackets. The curves are the Poisson regressions fitted to the data: *y *= *e^a+bt^*.

The variation of female fertility (number of perithecia produced by the evolved strains) and of male fertility (number of perithecia induced by the evolved strains but produced by the reference strains) with AG, monitored for S1, S2, S3 and S4 (two replicates, A and B) is presented in Figure 2. The decreases in female fertility and male fertility were both well fitted by Poisson distributions (*y *= *e^a+bt^*). For female fertility, the "intercept" (*a*), the "slope" (*b*) and the time after which the number of perithecia was halved (*t_50_*) are given in Table 1. We observed a complete loss of female fertility in all strains and replicates, except for replicate A of strain S1. For this evolved strain, a slight decrease was observed in the first AG, but female fertility increased again thereafter and never reached zero. The experiment lasted only 10 AGs for this replicate (vs 20 AG for replicate B), and this duration may have been insufficient for this strain to evolve towards female sterility. *t_50 _*reflects the speed at which female fertility was lost, so it was not calculated for S1-A. For S1-B, no perithecia were detected after 12 AGs of experimental evolution. For all the other strains, female fertility was completely lost in 10 to 19 AGs, in both replicates. The time until complete loss of female fertility varied between strains and between replicates. In S2, female fertility was lost in 19 AGs in replicate A and 12 AGs in replicate B. In S3, female fertility was lost in 10 AGs for replicate A and in 15 AGs for replicate B. In S4, female fertility was lost in 19 AGs in replicate A and 10 AGs in replicate B. ANOVA revealed significant effects of mating type and replicate on *t_50 _*(Table 3). Thus, almost all the strains lost female fertility, but the rate of loss was different for the two replicates of the strain and between different mating types.

**Table 3:** ANOVA of *t_50 _*for loss of female fertility as a function of mating type, strain, replicate and reference strain used for crosses

Source of variation	df	Sum of squares	Mean Square	*F *values	*P *values
Mating type	1	12.4	12.4	12.5	0.017 *
Wild-type strain	2	7.4	3.7	3.7	0.103
Replicate	3	31.1	10.4	10.4	0.014 *
Reference strain	2	6.3	3.1	3.1	0.130
Residuals	5	5.0	1.0		

*df*: degrees of freedom. * significant at the 5% level

As we did not carry out any single-spore isolation, intermediate AGs are expected to be mixtures of female-fertile and female-sterile individuals. We checked this by isolating 10 single-spore strains at the fifth AG of experiment A for each evolved strain. We then assessed the female fertility of these single-spore strains. There were 3, 5, 1, and 2 female-sterile single-spore strains for S1-A_5_, S2-A_5 _S3-A_5_, and S4-A_5_, respectively. Thus, as intermediate AGs are mixtures of female-fertile and female-sterile individuals, the number of perithecia in each AG probably reflects the proportion of female-fertile strains in the mixture. This result also confirms that female-sterile strains appeared relatively early in the experiment (before the 5th AG).

For two strains, another phenotypic modification was observed during the experiment. In S2, in both replicates, perithecia were found all over the plate and not just at the line of contact between the different mycelia at the 9th AG. This phenotype disappeared from both replicates at the 10th AG, but reappeared in replicate A between the 12th and the 15th AG (corresponding to a peak in the number of perithecia in Figure 2). The same phenotype was observed in replicate A of S4 at the 15th AG. These perithecia contained no ascospores. This phenotype may reflect the autoformation of sterile perithecia, as already reported for finger millet strains of *M. oryzae *after subculturing [34]. At the end of the experiment, these strains became female-sterile and male-sterile. We checked that the fitted Poisson regressions remained unchanged when measurements at AGs with autoformed perithecia were removed from the datasets. With this correction, the regressions had higher *R^2 ^*values, but the values of the parameters differed only slightly (data not shown).

### Decrease in male fertility

Male fertility was also affected during the experiment. Poisson regressions were also fitted to the number of perithecia produced by the reference strains when crossed with the evolved strains. At the end of the experiment, male fertility was completely lost in about half the replicates: S1-B, S2 (replicates A and B), S3-B and S4-A. We investigated the correlation between changes in female fertility and male fertility over time, by carrying out Pearson's correlation tests to compare the number of perithecia produced with the number of perithecia in the reference strains induced by the evolved strains at different AGs. Positive significant correlations were found, but neither for all strains nor for all replicates (Table 1). Male fertility and female fertility were positively correlated for both replicates of S4, in crosses with both reference strains. A positive significant correlation was also found for S2, but only for replicate B in crosses with both references. The correlation was also significantly positive for S3, for both replicates, but only in crosses with reference strain S2. There was no significant correlation between male fertility and female fertility for S1 replicates A and B in crosses with both references. It was therefore not possible to conclude that there was a general correlation between male and female fertility over time.

### Do the evolved female-sterile phenotypes have an epigenetic or genetic basis?

As female fertility was frequently and relatively rapidly lost in our experiment, we initially suspected a role for epigenetic control. Phenotypic modifications controlled by epigenetic mechanisms can be reversed by the reprogramming of gene expression following stresses or developmental processes [40,41,43]. We thus addressed the question of possible epigenetic control of female sterility by subjecting the female-sterile evolved strains S1-B_12 _and S3-A_10 _to various stresses (cold, mycelium fragmentation) and developmental processes (formation of conidia, host infection) and determining whether these stresses could restore the wild-type female-fertile phenotype. As control, we also subjected the wild-type strains S1 and S3 to the same stresses and assessed their female fertility. Whatever the treatment imposed, the two wild-type strains remained female-fertile, confirming that the chosen treatments did not affect female fertility. None of the treatments or cellular events tested (including meiosis, see below) reversed the female-sterile phenotype of the evolved strains, S1-B_12 _and S3-A_10 _(Table 2). We cannot rule out the possibility of epigenetic control, but these results support the hypothesis of a genetic origin of the experimental loss of female fertility.

We then tested the genetic hypothesis by performing crosses between female-sterile evolved strains and wild-type strains, to determine whether the evolved phenotype segregated in the offspring and to determine the number of genes involved in the loss of female fertility (Table 4). As expected, all the offspring of the control cross between the two wild-type female-fertile strains S1 and S3 (cross #125) were female-fertile. In the offspring of the cross between S1 and S3-A_10 _(cross #126), the ratio of female-fertile:female-sterile strains was significantly different from 1:1 (one gene; *χ*^2 ^= 7.53, *P *= 0.006, df = 1) but not significantly different from 1:3 (two genes; χ^2 ^= 1.13, *P *= 0.29, df = 1). Backcrosses were also performed between a Mat1.1 female-sterile progeny (126/0/4) and the wild-type strain S3 (cross #130) and between a Mat1.2 female-sterile progeny (126/0/35) and the wild-type strain S1 (cross #133). In the offspring of cross #130, the ratio was also not significantly different from 1:3 (*χ*^2 ^= 0.01, *P *= 0.92, df = 1). In the offspring of cross #133, the observed ratio did not significantly depart from either 1:1 or 1:3 (*χ*^2 ^= 2.78, *P *= 0.10, df = 1, and *χ*^2 ^= 2.27, *P *= 0.12, df = 1, respectively). These results suggest that the loss of female fertility in S1-A_10 _is controlled by two independent genes. However, the data could also support the hypothesis of one gene with segregation distortion. In the offspring from the cross between S1-B_12 _and S3 (cross #127), the ratio of female-fertile:female-sterile strains was significantly different from 1:1 but not significantly different from 1:3 (*χ*^2 ^= 0.30, *P *= 0.80, df = 1). Backcrosses were also performed between a Mat1.2 female-sterile progeny (127/0/25) and the wild-type strain S1 (cross #131) and between a Mat1.1 female-sterile progeny (127/0/28) and the wild-type strain S3 (cross #132). The ratio was not significantly different from 1:3 in cross #132 (*χ*^2 ^= 0.01, *P *= 0.93, df = 1). In cross #131, the observed ratio departed from 1:1 (*χ*^2 ^= 8.40, *P *= 0.004) and from 1:3 (*χ*^2 ^= 50.9, *P *< 0.001, df = 1), but was not significantly different from 3:1 (*χ*^2 ^= 0.19, *P *= 0.66, df = 1). The results of cross #127 and backcross #132 again suggest that the loss of female fertility in S1-B_12 _is controlled by two independent genes. The segregation observed in backcross #131 could be explained by other hypotheses (e.g. one gene with distortion, geno-cytoplasmic control,...) but additional crosses would be required to test these hypotheses. Nevertheless, for the two mutants analyzed, the segregations of female sterility in the offspring of the first generation cross and in backcrosses support a genetic control of sterility rather than epigenetic control.

**Table 4:** Segregation of mating type and female fertility in the progenies of crosses between female-sterile evolved strains S1-B_12 _and S3-A_10 _and wild-type strains S3 and S1, and in backcross progenies

					**Observed**	**segregation**			**Mat1:Mat2**		**f:s**		
							
**Cross number**	**Parental**	**strains**	**Parental**	**phenotypes**	**Mat1.1-f**	**Mat1.2-f**	**Mat1.1-s**	**Mat1.2-s**	**Observed**	**Chi^2 ^and P (1:1)**	**Observed**	**Chi^2 ^and P (1:1)**	**Chi^2 ^and P (1:3)**
		
	S1	S3	Mat1-f	Mat2-f	21	18	0	0	21:18	0.23 (0.631)	39:00		
126	S1	S3-A10	Mat1.1-f	Mat1.2-s	**6**	3	15	**10**	21:13	1.88 (0.170)	12:24	7.53 (0.006)	**0.04 (0.998)**
130	S3	126/0/04	Mat1.2-f	Mat1.1-s	7	**2**	**11**	15	18:17	0.03 (0.866)	09:26	8.26 (0.004)	**0.01 (0.922)**
133	S1	126/0/35	Mat1.1-f	Mat1.2-s	**9**	4	8	**15**	17:19	0.11 (0.739)	13:23	**2.78 (0.096)**	**2.27 (0.124)**
127	S3	S1-B12	Mat1.2-f	Mat1.1-s	5	**2**	**15**	15	20:17	0.24 (0.622)	07:30	14.3 (0.000)	**0.73 (0.866)**
131	S1	127/0/25	Mat1.1-f	Mat1.2-s	**13**	18	6	**6**	19:24	0.58 (0.446)	31:12	8.40 (0.004)	50.9 (0.000)
132	S3	127/0/28	Mat1.2-f	Mat1.1-s	4	**6**	**18**	11	22:17	0.64 (0.423)	10:29	9.26 (0.002)	**0.01 (0.926)**

*f*: female-fertile, *s*: female-sterile (mutant). The control cross is the cross between the two wild-type strains S1 and S3 (first line). Values in bold indicate non significant differences at the 5% level

We were unable to determine whether the genes responsible for female sterility were identical in the different mutants by classical allelism tests, because these mutants were sterile and therefore could not be crossed.

### Fitness comparisons between evolved and wild-type strains

As female-sterile mutants replaced female-fertile strains, we reasoned that the mutants must have a fitness advantage. We focused on traits relating to vegetative growth and asexual multiplication, because this mode of reproduction was the only one operating during the evolution experiment. We used several different approaches to address this point.

#### Measurement of fitness traits in female-fertile wild-type and female-sterile evolved strains grown separately

We first compared several traits potentially involved in fitness between wild-type and evolved strains grown separately. We focused on three particular traits relating to asexual reproduction: the rate of vegetative growth, the rate of asexual sporulation (*in vitro *and *in planta*) and the number of asexual conidia transferred *in vitro *at the end of an AG.

The rate of mycelial growth was assessed by determining the area of the Petri dish covered by the mycelium after seven days. This area was significantly smaller in S3-A_10 _than in S3 (Kruskall-Wallis *χ*^2 ^= 6.81, *P *= 0.009, df = 1), but did not differ significantly between S1-A_12 _and S1 (Kruskall-Wallis *χ*^2 ^= 1.84, *P *= 0.17, df = 1).

**Figure 3:**
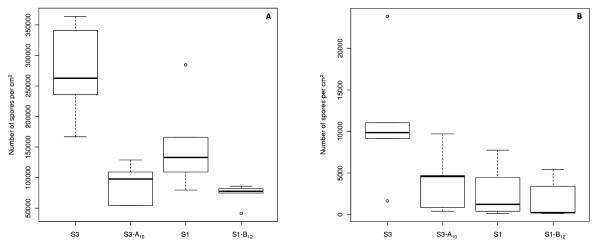
**Boxplots of number of conidia per cm^2 ^produced by the evolved strains S1-B_12 _and S3-A_10 _and the corresponding wild-type strains S1 and S3, respectively**. Conidial production was assessed in vitro (A) and on rice plants (B).

We then compared the production of conidia *in vitro *between evolved strains S1-B_12 _and S3-A_10 _and their corresponding wild-type strains, S1 and S3 (Figure 3A). In the evolved strain S3-A_10_, levels of *in vitro *asexual sporulation were significantly lower, at only one quarter those in the corresponding wild-type strain S3 (Kruskall-Wallis *χ*^2 ^= 6.82, *P *= 0.009, df = 1). This was also true for evolved strain S1-B_12_, for which asexual sporulation rates were half those of the corresponding wild-type strain S1 (Kruskall-Wallis *χ*^2 ^= 4.81, *P *= 0.028, df = 1). For the other evolved strains that had lost female fertility (data not shown), despite the use of only one or two replicates, we also observed a trend towards a decrease in asexual sporulation (rates for S3-B_15 _one fifth those for S3, rates for S2-A_19 _and for S2-B_12 _lower than those for S2 by factors of 1.5 and 2, respectively, and rates for S4-B_10 _lower than those for S4 by a factor of 1.5). This trend was not observed for S4-A_19_. Overall, these results show that, at least *in vitro*, female-sterile mutants are either unaffected or differ from the corresponding female-fertile wild-type strains in their vegetative growth and capacity to produce asexual spores.

Previous studies on mutants with altered sporulation reported opposite results for sporulation between measurements *in vitro *and *in planta *[34]. We thus compared *in planta *asexual sporulation between S1-B_12 _and its wild-type strain S1, and S3-A_10 _and its wild-type strain S3. We also counted the number of lesions and measured their size seven days after plant inoculation. Lesion size was not significantly different, either between S1 and S1-B_12 _or between S3 and S3-A_10 _(P = 1 and P = 0.084, respectively). The number of lesions was significantly lower in S3-A_10 _than in S3 (38% lower, one-way ANOVA, *P *= 0.027). It was not significantly different between S1 and S1-B_12_, despite being 36% lower (one-way ANOVA, *P *= 0.051). Such differences may be accounted for by a lower level of conidial germination or appressorium formation. However, *in vitro*, no significant difference was found between S1 and S1-B_12 _or between S3 and S3-A_10 _for these processes (data not shown). No significant difference in asexual sporulation was observed *in planta *for any of the pairs compared (S1-B_12 _/S1: Kruskall-Wallis *χ*^2 ^= 0.53, *P *= 0.464, df = 1; S3-A_10 _/S3: Kruskall-Wallis *χ*^2 ^= 3.15, *P *= 0.076, df = 1; Figure 3B). Thus, female-sterile evolved strains S1-B_12 _and S3-A_10 _displayed a change in their capacity to sporulate on artificial medium but not *in planta*. These results suggest that, despite having an unaffected asexual sporulation capacity *in planta*, female-sterile evolved strains S1-B_12 _and S3-A_10 _may have a lower capacity to infect rice plants. However, sporulation results obtained *in vitro *or *in planta *cannot account for the replacement of female-fertile strains by female-sterile strains in our *in vitro *experiments.

**Figure 4:**
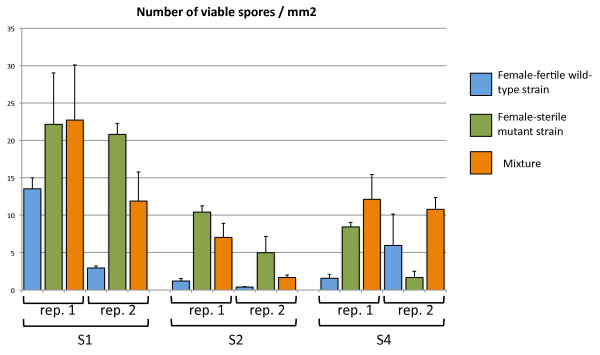
**Number of germinated conidia after transfer from one Petri dish to another (estimated over circles of 3 mm in diameter)**. Two independent replicates were generated. For each replicate, three areas on the plate were counted. The error bar represents the standard deviation calculated from these three counts. Blue bars: female-fertile wild-type strains (S1, S2 and S4). Green bars: female-sterile evolved strains (S1-B20, S2-A20 and S4-B20). Orange bars: mixtures of female-fertile wild-type strains and female-sterile evolved strains (S1+S1-B20, S2+S2-A20 and S4+S4-B20).

Finally, we determined whether the number of viable conidia (i.e. able to germinate) transferred between two AG was greater for female-sterile strains than for female-fertile strains (Figure 4). We found no significant difference in conidial germination rate between wild-type and mutant strains (99.2 ± 1.1 to 100 ± 0.0%, data not shown). However, the number of viable conidia transferred was greater for the female-sterile evolved strains than for the corresponding female-fertile wild-type strains. Three times as many viable conidia were transferred in female-sterile S1-B_20 _cultures (21.5 ± 1.0 conidia.mm^-2^) as in wild-type S1 cultures (8.3 ± 7.5 conidia.mm^-2^; Mann-Whitney U = 0.5, n1 = n2 = 6, *P *< 0.01). About 10 times as many viable conidia were transferred in the female-sterile S2-A_20 _cultures (7.7 ± 3.8 conidia.mm^-2^) as in the female-sterile S2 culture (0.8 ± 0.6 conidia.mm^-2^; Mann-Whitney U = 0, n1 = n2 = 6, *P *< 0.01). The number of viable conidia transferred was similar in the female-sterile S4-B_20 _cultures (5 ± 4.8 conidia.mm^-2^) and in the wild-type S4 cultures (3.7 ± 3.1conidia.mm^-2^; Mann-Whitney U = 16.5, n1 = n2 = 6, *P *> 5%). Thus, in two of the three pairs tested, female-sterile evolved strains transferred more conidia than their respective female-fertile wild-type strains. This result may explain the increasing predominance of female-sterile strains among evolved strains.

#### Measurement of fitness traits in female-fertile wild-type and female-fertile evolved strains in competition

We investigated whether the higher efficiency of conidial transfer constituted a fitness advantage in competition, by measuring this trait in competition experiments. In these experiments, the selective treatment was identical to that in the evolution experiment. In our protocol, it was not possible to distinguish between wild-type and mutant strains phenotypically when they were grown together. However, the ratio of viable conidia transferred from each strain at the end of an AG was measurable because, after their transfer, germinating conidia could be isolated and crossed with the appropriate reference strains to determine their fertility phenotype and hence their parental origin. S1-B_20 _was mixed with S1, S2-A_20 _with S2, S3-A_20 _with S3, and S4-B_20 _with S4. Single cultures of wild-type and mutant strains performed in the same conditions (see previous section) were used as controls. After one week, conidia were transferred to a new dish and counted as described above. The germination rate of conidia in mixtures (98.6 ± 2.0 to 99.8% ± 0.3; data not shown) was not different from the germination rate in single cultures (99.2 ± 1.1 to 100 ± 0.0%; data not shown). We determined the ratio of female-sterile to female-fertile strains in these transferred conidia, by isolating 20 germinated conidia per mixture and assessing their female fertility. For the S1 + S1-B_20 _mixture, the observed ratio (55%) was not significantly different from the expected 50% ratio (*χ*^2 ^= 0.4, *P *= 0.522, df = 1; Table 5). For the S2 + S2-A_20 _and S3 + S3-B_20 _mixtures, 97.5% of the strains recovered were female-sterile (*χ*^2 ^= 36.1, *P *= 0.000, df = 1 for both mixtures). For the S4 + S4-A_20 _mixture, 70.0% of the strains recovered were female-sterile, a ratio significantly greater than the expected 50% ratio (*χ*^2 ^= 6.4, *P *= 0.011, df = 1). These results show that, in competition, the female-sterile mutants of S2, S3 and S4 transferred conidia more efficiently that the female-fertile wild-type strains from which they were derived.

**Table 5:** Ratio of female-sterile and female-fertile strains transferred in a single asexual generation from mixtures of female-sterile and female-fertile strains

	No. of female-sterile strains recovered/20 strains		Mean % of female-sterile strains recovered	Comparison with expected 50% ratio	Expected % based on conidial transfer evaluation in single culture
				
Mixture	Rep1	Rep2			
S1 + S1B20	10	12	55	NS (Chi^2 ^= 0.4, *P *= 0.522)	72.1
S2 + S2A20	19	20	97.5	S (Chi^2 ^= 36.1, *P *= 0.000)	90.6
S3 + S3B20	19	20	97.5	S (Chi^2 ^= 36.1, *P *= 0.000)	nd
S4 + S4A20	13	15	70	S (Chi^2 ^= 6.4, *P *= 0.011)	57.5

The expected percentage based on conidial transfer evaluation in a single culture was calculated by averaging the observed number of conidia transferred for each of the two strains when grown individually

## Discussion

We demonstrate here, for the first time, that female fertility can be lost *in vitro *in *M. oryzae *rice strains grown asexually. This loss could be observed in 10 to 19 asexual generations, representing at least 20 000 to 38 000 mitotic generations. All strains tested displayed a loss of female fertility for at least one replicate. The loss of fecundity in laboratory conditions has already been documented in other fungal species, such as *Blastomyces dermatidis *and *Histoplasma capsulatum *[15]. The specific loss of female fertility has also been described before, in an *M. oryzae *strain from finger millet [34], in which the female-sterile phenotype was found to be controlled by a single gene. In our experiment, the female-sterile phenotype that appeared during the experiment segregated in the first-generation crosses and in backcrosses. This result is consistent with the hypothesis that the loss of female fertility is probably due to genetic mechanisms, such as mutations, gene deletion or major chromosomic rearrangements [17], rather than epigenetic mechanisms. We were expecting a 1:1 segregation of female-fertile:female-sterile strains in the offspring, indicative of the involvement of a single gene. The 1:3 ratio observed in most crosses suggests the involvement of two genes. Independent mutations in two unlinked genes would have occurred twice, once in strain S1-B_12 _and once in strain S3-A_10. _However, we cannot rule out the possibility of mutations in a single gene and distortion of the segregation of this gene. Additional crosses would be required to distinguish between these two hypotheses. Segregation distortion has been documented in many Eukaryotes, sometimes resulting in a bias in sex ratio [44,45]. Theoretical studies suggest that segregation distortion may play an important role in the evolution of sexual reproduction, acting, for instance, on sex determination [45]. Allelism tests would be a suitable method for determining whether the same locus is affected in all strains and all replicates. However, it is not possible to cross two female-sterile strains. Genetic mapping of the mutations would make it possible to compare gene positions and to determine whether different loci were involved. Alternatively, complementation experiments could be performed by smashing the mycelia of two female-sterile evolved strains together to restore female fertility. We performed this experiment with two strains (S2-A_5 _and S4-A_5_) after the single-spore isolation of female-sterile conidia, but no restoration of female fertility was observed. We could perform complementation experiments on all combinations of pairs of female-sterile evolved strains, to test the hypothesis that there are several mutations at different loci.

We provide the first demonstration of the loss of such an important life-history trait as female fertility in *M. oryzae *rice strains. In an experiment on *M. oryzae *on artificial medium and on plants over 10 generations, Park *et al. *[46] observed no change in the pathogenicity or genome sequences of avirulence genes. They concluded that the genome was highly stable. However, each generation resulted from the transfer of only one or two conidia after two weeks of growth. So, even if mutants appeared at a normal rate, the sample studied by Park was too small for their detection. By contrast, we observed a gradual decrease in perithecium production until complete female sterility was attained. This may be explained by the transfer of at least of 27,000 (± 24,000) conidia -- constituting a population -- between Petri dishes at each generation. We therefore observed the process by which female-sterile mutants occur and colonise within a population. Consequently, the number of perithecia reflected the proportion of wild-type female-fertile individuals remaining in the population. Monospore isolation at an intermediate generation confirmed that the evolved strains consisted of mixtures of female-fertile and female-sterile individuals. The transfer of several thousands of conidia between asexual generations resulted in a smaller bottleneck than the transfer of single spores and favoured invasion by mutants and, thus, their detection.

The detection of female-sterile mutants was also probably favoured because female-sterile mutants had a selective advantage, allowing them to increase in the population. We first checked that the mycelium of the female-sterile mutants did not induce necrosis of the mycelium of female-fertile strains when grown together (data not shown). Fitness differences were revealed by several experiments in which traits relating to fitness were compared between female-fertile and female-sterile strains grown either individually or in competition. These differences in fitness were not accounted for by differences in growth rates, because growth rates (measured individually) were no higher for female-sterile than for female-fertile strains, either *in vitro *or *in planta*. For one of the four mutants tested, grown either separately or in competition in the exact selective conditions of the evolutionary experiment, we detected no selective advantage. This may be related to complex dynamics of fitness gain [27]. However, for the other three mutants, we showed that the conidia of female-sterile mutants were transferred significantly more efficiently than those of female-fertile strains. This greater efficiency of transfer was probably linked to differences in the conidial attachment process. This alteration favoured the release of conidia from female-sterile mutants over that of conidia from wild-type strains. This unexpected change in asexual reproduction phenotype may account for the dynamics of the loss of sexual reproduction observed in our study: the decrease in the number of perithecia, reflecting the invasion of the population by female-sterile mutants, was initially rapid and then gradually slowed down until female fertility was completely lost.

We also showed that the loss of female fertility, and hence of sexual reproduction, was associated with modifications to asexual spore production *in vitro*. We expected to see a trade-off between sexual and asexual reproduction, in the form of an increase in asexual spore production with decreasing female fertility. However, we actually observed the opposite: asexual sporulation rates were lower in experimental strains that had lost female fertility, at least *in vitro*, and also, indirectly, *in planta*, due to a decrease in the number of lesions produced after infection. This finding can be accounted for by independent mutations or by mutations of a single locus with positive pleiotropic effects on sexual and asexual reproduction. The existence of a positive correlation between sexual and asexual reproduction capacity has already been observed in various pathosystems. For example, in an experiment on a finger millet strain of *M. oryzae*, Tharreau *et al. *[34] showed that female sterility was associated with higher levels of asexual sporulation *in vitro *but lower levels of sporulation *in planta*. Ali *et al. *[47] found a positive correlation between sexual spore production and asexual spore production. Zeyl *et al. *[48] found a positive correlation between sexual fitness and asexual fitness in wild-type strains in *Saccharomyces cerevisiae *when sexual selection was applied. A decrease in asexual reproduction capacity associated with a decrease or even a loss of sexual reproduction capacity *in vitro *has also been observed in *Cryptococcus neoformans *[49]. Hill and Otto [50] showed positive pleiotropic effects between sexual sporulation and asexual sporulation in the experimental evolution of *S. cerevisiae*. Mycelial growth rate and sporulation rate are often used to assess fitness in fungi [26]. However, we found, in the progenies of two crosses, that the decrease in asexual sporulation was independent of female fertility (data not shown). This study therefore provides no evidence of a pleiotropic effect of mutations conferring sterility on asexual sporulation rate.

Another interesting result was the decrease in male fertility. About half the eight strains had completely lost the ability to induce the production of perithecia by another strain by the end of the experiment. The correlation between the decrease in male fertility and the loss of female fertility observed for some of the evolved strains cases may reflect pleiotropic effects of mutations.

We also found that, at certain times, evolved strains were able to produce perithecia in the absence of strains of the opposite mating type, but this trait was not observed at all time points. The autoformation of sterile perithecia has already been reported in one *M. oryzae *isolate from finger millet [34] and the gene responsible for female fertility has negative epistatic effects on the gene responsible for the autoformation of sterile perithecia. The occurrence of such a phenotype may also result from mutants that rapidly died out due to their lower fitness.

As female fertility and sexual reproduction seem to be easily lost, it remains unclear why some *M. oryzae *strains have remained female-fertile in Asia. Sexual reproduction presumably confers some advantage on these strains in this environment. In some fungi, organs of sexual reproduction constitute resting structures allowing the fungus to survive in adverse conditions. However, *M. oryzae *ascospores have a short lifetime and perithecia are thus unlikely to constitute a resting stage *in natura*. As Asia is the centre of domestication of rice [51], the varieties in this area are likely to be more diverse than elsewhere in the world. In such a heterogeneous environment, there may be selection for strains able to reproduce sexually [31,52].

## Conclusion

We showed, through experimental evolution, that female fertility could be lost in *M*. oryzae and that this loss involved genetic mechanisms rather than epigenetic mechanisms. The experimental evolution of microorganisms is a suitable method for investigating different evolutionary mechanisms [53], including those potentially involved in the loss of sexual reproduction in several species [54]. Experimental evolution *in vitro *does not strictly reflect what happens *in natura*, but can be viewed as an approximation of a situation in a favourable constant environment [55]. We show here that female fertility was lost easily after only a few cycles of strictly asexual reproduction. This study provides new insight into the processes underlying the evolution of mode of reproduction in *M. oryzae in natura*. However, recent studies have shown that although *M. oryzae *populations are strictly asexual and female-fertile strains are absent from most rice-growing areas [33], this species continues to reproduce sexually in its centre of origin in Asia, at least over limited areas [31]. We previously suggested that the ability to reproduce sexually was lost during the spread of the pathogen from its centre of origin to the rest of the world, accompanying the host-tracking process during rice domestication and dissemination. This loss may have resulted from genetic drift after a bottleneck during the introduction of the fungus into a new area. If a single mating type was introduced into a new habitat, sexual reproduction would not have been possible and the fungus would have been forced to reproduce asexually. The results of our study suggest that, under these conditions, female fertility could have been lost very rapidly (one epidemic season), leading to a complete and definitive loss of the ability to reproduce sexually.

Genetic mutations distinguishing between evolved and ancestral genotypes must be identified to disentangle the genetic bases of the phenotypic modifications observed in our experiment. A combination of genetic studies and high-throughput sequencing methods would potentially speed up identification of the genes responsible for female fertility/female sterility. Studies of the evolution of polymorphisms of these genes between natural female-fertile, sexually reproducing strains from Asia and natural female-sterile, clonally reproducing strains from other parts of the world would then improve our understanding of the evolution of modes of reproduction in *M. oryzae*.

## Authors' contributions

DS, DT and EF designed the study. DS carried out the experiment, analyzed the data, interpreted the results and drafted the manuscript. JM and HA contributed to the laboratory work. DT contributed to the laboratory work, the data analyses, the interpretation of the results, and to manuscript writing. EF contributed to the laboratory work, the data analyses, the interpretation of results, and to manuscript writing. All the authors have read and approved the final manuscript.
